# Targeting Inflammation in Chronic Kidney Disease: Pathophysiological Insights and Emerging Therapeutic Strategies

**DOI:** 10.3390/jcm15176550

**Published:** 2026-08-25

**Authors:** Aris Tsalouchos, Pietro Claudio Dattolo

**Affiliations:** Nephrology and Dialysis Unit Firenze 2, Department of Medical Specialties, Azienda USL Toscana Centro, 50100 Firenze, Italy; pietroclaudio.dattolo@uslcentro.toscana.it

**Keywords:** chronic kidney disease, inflammation, innate immunity, NLRP3 inflammasome, interleukin-6, cardiovascular risk, SGLT2 inhibitors, precision medicine

## Abstract

Chronic kidney disease (CKD) is sustained by a network of sterile inflammation, oxidative and metabolic stress, uremic toxin retention, gut barrier dysfunction, and maladaptive immune activation. These processes contribute to kidney fibrosis, cardiovascular injury, wasting, and excess mortality, but inflammatory biomarkers do not by themselves establish therapeutic causality. This narrative review integrates mechanistic and therapeutic evidence using an explicit three-layer translational hierarchy. Renin–angiotensin system inhibitors, sodium–glucose cotransporter-2 inhibitors, finerenone, and glucagon-like peptide-1 receptor agonists improve cardiorenal outcomes and have plausible anti-inflammatory actions, although inflammatory mediation remains unproven. Interleukin-1 blockade provides cardiovascular proof of principle and small dialysis feasibility data. Interleukin-6 ligand inhibition produces marked human target engagement; however, headline results from the completed phase 3 ZEUS trial showed no reduction in three-point major adverse cardiovascular events with ziltivekimab despite biomarker suppression, while serious infections were more frequent. POSIBIL6ESKD continues to test clazakizumab in inflamed dialysis patients. Direct NLRP3 inhibition has entered early human CKD development, whereas senescence-directed and microbiota-based approaches remain less mature. Future progress requires inflammatory endotyping, repeated biomarker assessment, mechanistically aligned outcomes, and rigorous infection surveillance. ZEUS underscores that pathway suppression must deliver clinical benefit beyond contemporary standard therapy.

## 1. Introduction

Chronic kidney disease (CKD) has become a defining non-communicable disease of the twenty-first century. The Global Burden of Disease 2023 analysis estimated that 788 million adults were living with CKD in 2023, corresponding to an age-standardized prevalence of 14.2%. CKD was the ninth leading cause of death worldwide, directly accounting for 1.48 million deaths, while impaired kidney function contributed to an estimated 11.5% of cardiovascular deaths [1]. Complementary national analyses illustrate marked geographic heterogeneity; in China, the number of people living with CKD more than doubled between 1990 and 2023, with diabetes and hypertension accounting for an increasing share of disease burden [2]. Despite this burden, CKD is still frequently conceptualized as progressive nephron loss driven primarily by hemodynamic and metabolic injury. Contemporary evidence supports a broader view: CKD is also a systemic inflammatory disorder in which kidney injury, maladaptive immunity, vascular pathology, and impaired host defense coexist and reinforce one another [3,4,5,6,7,8,9].

This reframing is clinically relevant. Renin–angiotensin system (RAS) blockade, sodium–glucose cotransporter-2 (SGLT2) inhibitors, nonsteroidal mineralocorticoid receptor antagonism, and glucagon-like peptide-1 receptor agonists (GLP-1RAs) have substantially improved cardiorenal outcomes. Nevertheless, residual risks of kidney failure, heart failure, atherosclerotic events, infection, and death remain high, particularly in advanced CKD and dialysis [3,4,5,6,7,8,9]. Inflammation is an attractive candidate contributor to this residual risk because it is biologically connected to fibrosis, endothelial dysfunction, atherothrombosis, calcification, anemia, protein-energy wasting, and immune dysfunction.

The therapeutic inference is not straightforward. Elevated high-sensitivity C-reactive protein (hsCRP), interleukin (IL)-6, soluble tumor necrosis factor receptors, or other inflammatory markers may identify patients at high risk without proving that the measured pathway is causal or safely modifiable. Conversely, drugs that improve CKD outcomes may lower inflammation as a downstream consequence rather than through the mechanism responsible for benefit. A clinically useful review must therefore distinguish association from causality, biomarker reduction from disease modification, and preclinical promise from outcome-proven therapy.

The aim of this narrative review is to integrate the inflammatory biology of CKD with the current therapeutic landscape while preserving this evidentiary distinction. We first describe the principal upstream drivers and signaling hubs, then examine how they contribute to renal and cardiovascular complications. We subsequently evaluate established and emerging interventions according to translational maturity and conclude with a precision-medicine framework for clinical trials and future implementation.

## 2. Review Design, Literature Identification, and Evidence Framework

### 2.1. Review Type and Information Sources

This article is a targeted narrative review rather than a systematic or scoping review. Its purpose is to provide a critical translational synthesis of mechanistic biology, outcome-proven cardiorenal therapy, and pathway-directed anti-inflammatory development in CKD. PubMed/MEDLINE and ClinicalTrials.gov were searched until July 2026. Separate, reproducible search strategies for each source are reported in Supplementary Methods S1. Reference lists of key guidelines, randomized trials, and recent high-quality reviews were also screened, and registry identifiers were cross-checked immediately before revision.

### 2.2. Eligibility, Screening, and Study Selection

Eligible therapeutic evidence comprised adult studies enrolling participants with non-immune CKD, kidney failure receiving maintenance dialysis, or CKD-enriched cardiovascular disease; interventions had to directly target an inflammatory pathway or be an established cardiorenal therapy with a clinically relevant anti-inflammatory mechanism. Priority was given to guidelines, randomized outcome trials, randomized human target-engagement studies, systematic reviews, and prospectively registered interventional trials. Preclinical studies were retained only for pathways without CKD-specific human efficacy data. Disease-specific immunosuppression for active glomerulonephritis, transplantation, and acute kidney injury; isolated biomarker association studies; and trials conducted entirely outside CKD without direct translational relevance were excluded from the therapeutic pipeline.

Both authors reviewed candidate publications and registry records and reached final inclusion decisions by consensus. No formal duplicate risk-of-bias assessment, quantitative meta-analysis, or exhaustive study-count flow diagram was undertaken because selection was purposive and aligned with a narrative review. All registered interventional studies meeting the prespecified pipeline criteria and identified at the updated search are included in the therapeutic pipeline and detailed in Supplementary Table S1.

### 2.3. Operational Translational Hierarchy

Therapeutic evidence was interpreted across three operational layers. Layer 1 requires benefit on a prespecified kidney or cardiovascular clinical outcome in an adequately powered randomized CKD or CKD-enriched trial; an anti-inflammatory mechanism may be plausible but need not be proven as the mediator. Layer 2 requires randomized human evidence in CKD of pathway engagement, biomarker modulation, or a validated surrogate response, but lacks demonstrated hard-outcome benefit. Layer 3 comprises preclinical efficacy, first-in-human safety/pharmacodynamic programs, or small heterogeneous clinical studies without reproducible CKD efficacy. Movement from Layer 3 to Layer 2 requires human target engagement with acceptable safety, and movement from Layer 2 to Layer 1 requires a favorable clinical outcome trial rather than biomarker suppression alone. Movement is not necessarily unidirectional: neutral or harmful outcome results constrain a program even when target engagement is robust, as illustrated by ZEUS and BEACON.

### 2.4. Methodological Limitations

Several methodological limitations should be considered. This is a targeted narrative review rather than a systematic or scoping review, and no protocol was prospectively registered. Study identification and inclusion were therefore purposive. Searches were limited to PubMed/MEDLINE and ClinicalTrials.gov and did not systematically cover Embase, Web of Science, Scopus, the Cochrane Library, preprint servers, or conference proceedings. The English-language restriction may have excluded relevant evidence published in other languages. Although both authors reviewed candidate publications and registry records and reached inclusion decisions by consensus, duplicate independent screening, a standardized data-extraction form, and a formal study-level risk-of-bias or certainty assessment were not undertaken. These features increase the possibility of selection, language, publication, and reporting bias and limit reproducibility compared with a systematic evidence synthesis.

The therapeutic pipeline is particularly sensitive to incomplete and rapidly changing information. ClinicalTrials.gov records can be modified after the search date, may contain inconsistent enrollment or status fields, and may not provide results for completed studies; trials registered exclusively in other registries, incompletely indexed studies, and unpublished programs may consequently have been missed. Sponsor communications were used only for time-sensitive trial status or headline findings and were explicitly distinguished from peer-reviewed evidence; nevertheless, such reports provide less methodological detail and may be affected by selective outcome reporting. In addition, heterogeneity in CKD cause and stage, dialysis status, inflammatory enrichment, background therapy, biomarker definitions, follow-up duration, and clinical endpoints precluded quantitative comparison across interventions.

Finally, the three translational layers constitute an interpretive framework proposed by the authors rather than a validated evidence-grading system. Layer assignment may change as full trial reports, longer-term safety data, and additional outcome studies become available, and grouping therapies within a common layer can obscure important differences in mechanism, dose, target population, and endpoint hierarchy. Biomarker or surrogate responses cannot establish causal mediation or net clinical benefit; conversely, a neutral trial in one clinical phenotype does not exclude benefit in a more precisely selected population. Accordingly, this review should be interpreted as a transparent, time-limited critical synthesis of evidence identified through July 2026, with registry status rechecked on 16 August 2026, rather than as an exhaustive or formally graded evidence inventory.

## 3. The Inflammatory Architecture of CKD

### 3.1. Sterile Danger Sensing and Innate Immune Activation

Innate immunity is a principal interface between kidney injury and chronic inflammation. Pattern-recognition receptors identify pathogen-associated molecular patterns during infection and damage-associated molecular patterns (DAMPs) released by stressed or dying cells. In CKD, tubular epithelial cells, podocytes, endothelial cells, fibroblasts, resident macrophages, and circulating myeloid cells are repeatedly exposed to endogenous ligands such as high-mobility group box 1, extracellular ATP, mitochondrial DNA, heat-shock proteins, urate, calprotectin, and extracellular matrix fragments [4,5,10,11].

Toll-like receptors (TLRs), particularly TLR2 and TLR4, activate MyD88-dependent and alternative signaling pathways that converge on NF-κB and interferon-regulatory programs. The resulting transcriptional response increases tumor necrosis factor (TNF), IL-6, IL-1 family cytokines, chemokines, adhesion molecules, and profibrotic mediators. This response is initially protective, but persistent danger signaling prevents normal inflammatory resolution. Monocyte recruitment, macrophage polarization, tubular injury, and fibroblast activation then become components of a self-reinforcing tissue circuit rather than a transient response to damage [4,5,10,11].

### 3.2. The NLRP3 Inflammasome as an Integrative Hub

The NLRP3 inflammasome integrates structurally diverse signals that are abundant in CKD, including mitochondrial reactive oxygen species, potassium efflux, lysosomal injury, cholesterol and calcium-phosphate crystals, hyperglycemia, lipotoxicity, ischemia, and uremic toxins [12,13,14]. Canonical activation is commonly described as a two-step process. Priming increases transcription of NLRP3 and pro-IL-1β, often through NF-κB; a second signal promotes assembly of NLRP3 with the adaptor ASC and pro-caspase-1. Activated caspase-1 cleaves pro-IL-1β and pro-IL-18 and promotes gasdermin D-mediated pyroptosis [12,13,14].

This pathway is relevant across diabetic kidney disease, crystal nephropathies, immune-mediated glomerular injury, obstructive nephropathy, and tubulointerstitial fibrosis. NLRP3 may also exert inflammasome-independent effects on tubular apoptosis, mitochondrial function, and fibrotic remodeling [12,13]. However, the strength of evidence differs by context: genetic and pharmacological inhibition is consistently protective in experimental models, whereas human CKD data largely document pathway activation rather than clinical efficacy of direct inhibition. NLRP3 is therefore a biologically compelling target, but it remains in an early translational layer.

### 3.3. Cytokine Networks: IL-1, IL-18, and IL-6

IL-1β amplifies local leukocyte recruitment, endothelial activation, and profibrotic signaling, whereas IL-1α can function as an alarmin released from necrotic cells [15]. IL-18, produced by tubular and immune cells after inflammasome activation, has been implicated in hypertension, maladaptive repair, fibrosis, and cardiovascular injury [16]. These cytokines are interconnected rather than isolated: IL-1 promotes downstream IL-6 production, and both influence hepatic acute-phase responses and vascular inflammation.

IL-6 has particular translational importance because it connects renal injury with systemic consequences. Classical signaling through membrane-bound IL-6 receptor participates in host defense and regenerative responses, whereas trans-signaling through soluble IL-6 receptor can broaden pro-inflammatory effects across endothelial and other gp130-expressing cells [17]. In observational CKD cohorts, higher circulating IL-6 is consistently associated with cardiovascular events, CKD progression, and mortality [17,18]. In dialysis populations, systematic reviews likewise support an association between systemic IL-6 and all-cause and cardiovascular mortality, although between-study heterogeneity and non-standardized reporting limit precise risk estimation [19]. The central uncertainty is no longer whether IL-6 marks risk, but whether sufficiently selective and safe inhibition can modify that risk.

### 3.4. Immune Dysregulation Is More than Immune Activation

CKD produces the paradoxical combination of chronic inflammation and impaired antimicrobial immunity. Pro-inflammatory intermediate monocytes expand, endothelial adhesion increases, and monocyte/macrophage responses become metabolically reprogrammed [20]. At the same time, uremia is associated with dendritic-cell dysfunction, lymphocyte exhaustion, impaired vaccine responses, and susceptibility to infection. This coexistence is critical for treatment: indiscriminate immunosuppression may reduce inflammatory markers while worsening the outcome that patients with advanced CKD are already predisposed to experience.

Adaptive immunity also participates in CKD progression. Imbalances among effector T-cell subsets, regulatory T cells, B-cell signaling, and tissue-resident immune populations vary according to the primary kidney disease and stage. Such heterogeneity argues against a universal “inflammatory CKD” phenotype. A patient with active immune-complex glomerulonephritis, a patient with diabetic CKD and elevated hsCRP, and a patient receiving dialysis through an infected catheter may all have inflammation, but the dominant mechanisms and appropriate interventions are fundamentally different.

### 3.5. Oxidative Stress, Mitochondrial Injury, and Defective Resolution

Oxidative stress and inflammation form a reciprocal circuit. Mitochondrial dysfunction, NADPH oxidases, uncoupled nitric oxide synthase, and activated immune cells increase reactive oxygen species, while antioxidant defenses governed partly by nuclear factor erythroid 2-related factor 2 (Nrf2) are impaired [21,22]. Reactive oxygen species activate NF-κB, facilitate NLRP3 assembly, oxidize lipids and proteins, and injure podocytes, tubular cells, and endothelium. Inflammatory cytokines, in turn, impair mitochondrial function and increase pro-oxidant enzyme activity.

This circuit helps explain why oxidative stress is detectable before kidney failure and why it persists despite conventional dialysis. It also illustrates a translational hazard. Restoring a cytoprotective pathway can improve a laboratory measure or even alter creatinine-based eGFR without necessarily improving structural kidney outcomes. Therapeutic manipulation of Nrf2 therefore requires evaluation of fluid balance, blood pressure, albuminuria, cardiovascular safety, and measured kidney function rather than reliance on eGFR alone.

### 3.6. Uremic Toxins and the Gut–Kidney Axis

Protein-bound uremic toxins such as indoxyl sulfate, p-cresyl sulfate, and indole-3-acetic acid accumulate as kidney function declines and are incompletely removed by conventional dialysis [23,24,25]. These compounds activate the aryl hydrocarbon receptor and NF-κB signaling, increase oxidative stress, disrupt endothelial function, and modify immune-cell behavior. They should therefore be regarded as biologically active mediators, not merely filtration markers [23,24,25].

The gut is an upstream source of several of these solutes. CKD-associated dysbiosis includes expansion of proteolytic metabolic pathways, reduced generation of beneficial short-chain fatty acids, and altered bile-acid and tryptophan metabolism. Urea and other retained solutes disrupt epithelial tight junctions, facilitating translocation of microbial products and low-grade endotoxemia [23,24,25,26]. Experimental transfer studies support a causal contribution of dysbiotic microbiota to inflammation and fibrosis, but clinical translation remains incomplete. Diet, medications, geographic variation, residual kidney function, and dialysis modality all shape the microbiome, making a single “CKD microbiota signature” unlikely.

### 3.7. Dialysis-Related Amplification

Inflammation becomes particularly complex in kidney failure. Recurrent blood-membrane interaction, endotoxin exposure, vascular access infection, periodontal and other occult infection, extracellular volume excess, intravenous iron, bioincompatibility, and loss of residual kidney function may all contribute. In peritoneal dialysis, local peritoneal and systemic inflammatory processes may be partly dissociated. In hemodialysis, intradialytic complement and leukocyte activation can coexist with chronic systemic cytokine elevation.

The dialysis setting also exposes the central therapeutic trade-off most clearly. Patients with persistent hsCRP or IL-6 elevation have high cardiovascular risk, yet they also have high rates of bacteremia, hospitalization, impaired vaccine response, and frailty. Direct cytokine inhibition in this population must therefore demonstrate not only target engagement but also a favorable net clinical benefit.

## 4. From Renal Inflammation to Systemic Disease

### 4.1. Maladaptive Repair and Fibrosis

Inflammation promotes CKD progression when a regenerative response fails to resolve. Recurrent tubular injury activates macrophages, pericytes, fibroblasts, and endothelial cells; cytokines and DAMPs then sustain transforming growth factor-β, chemokine, and extracellular matrix programs. Hypoxia caused by capillary rarefaction and increasing diffusion distance further injures tubular cells, while matrix stiffness and cellular senescence reinforce inflammatory and profibrotic signaling. The relevant therapeutic target may therefore change over time: suppressing an initiating immune pathway in early disease may not reverse established scar in advanced CKD.

This temporal dimension is frequently underappreciated in trials. Albuminuria can respond rapidly to hemodynamic or anti-inflammatory intervention, whereas meaningful separation in eGFR slopes or kidney-failure events may require longer follow-up. Conversely, a short-term eGFR increase may reflect altered creatinine handling or glomerular dynamics rather than nephron preservation. Mechanistic trials should therefore align the biomarker, disease stage, and duration with the biological process being targeted.

### 4.2. Endothelial Dysfunction and Atherothrombosis

CKD-related cardiovascular risk is not fully captured by LDL cholesterol or conventional risk scores. Activated monocytes adhere more readily to endothelium, inflammatory cytokines impair nitric oxide bioavailability, and oxidative modifications render lipoproteins dysfunctional [4,5,6,7,20,27]. Carbamylated and oxidized lipoproteins promote foam-cell formation and vascular inflammation even when measured LDL cholesterol is not markedly elevated. IL-6-driven acute-phase signaling also increases fibrinogen, serum amyloid A, secretory phospholipase A2, and prothrombotic activity.

These mechanisms provide the rationale for cytokine-directed cardiovascular outcome trials in CKD. Importantly, such trials primarily test whether inflammation contributes causally to atherosclerotic and heart-failure outcomes in a CKD-enriched population; they do not automatically establish a direct effect on intrinsic kidney disease.

### 4.3. Vascular Calcification and Premature Aging

Vascular calcification in CKD is an active cell-mediated process. Hyperphosphatemia, oxidative stress, inflammatory cytokines, and uremic toxins promote osteogenic transdifferentiation of vascular smooth-muscle cells, extracellular-vesicle release, and loss of endogenous calcification inhibitors [28,29,30]. IL-1β, IL-6, and TNF interact with reactive oxygen species and mineral stress to accelerate this transition. Medial calcification, arterial stiffness, left ventricular afterload, and microvascular dysfunction then amplify cardiovascular risk.

CKD also resembles accelerated biological aging. Mitochondrial dysfunction, phosphate toxicity, Klotho deficiency, DNA damage, and chronic inflammation induce cellular senescence in endothelium, vascular smooth-muscle cells, podocytes, and tubular cells [31,32]. Senescent cells adopt a senescence-associated secretory phenotype rich in IL-6, chemokines, proteases, and profibrotic mediators. Senescence is therefore both a consequence and an amplifier of CKD inflammation, although senolytic and senomorphic strategies remain experimental.

### 4.4. Anemia, Wasting, Frailty, and Infection Vulnerability

Inflammation impairs iron mobilization through hepcidin, blunts erythropoietin responsiveness, promotes muscle catabolism, suppresses appetite, and lowers hepatic albumin synthesis. These processes link systemic inflammation to anemia, protein-energy wasting, and frailty. The resulting phenotype is prognostically adverse but difficult to dissect because comorbidity, occult infection, volume overload, and inadequate nutrition may produce similar biomarker patterns.

Inflammatory activation must also be interpreted alongside immune dysfunction. A fall in hsCRP after targeted therapy may be biologically desirable, but the clinically relevant result is the balance among cardiovascular events, kidney outcomes, serious infection, cytopenia, wound healing, and patient-reported function. This multidimensional endpoint framework is especially important in dialysis.

## 5. Therapeutic Strategies Across the Translational Continuum

### 5.1. Outcome-Proven Therapies with Pleiotropic Anti-Inflammatory Actions

#### 5.1.1. RAS Blockade

Angiotensin II promotes oxidative stress, NF-κB activation, endothelial dysfunction, and leukocyte recruitment. ACE inhibitors and angiotensin receptor blockers can reduce inflammatory markers in addition to lowering intraglomerular pressure and albuminuria [33]. Their established clinical role, however, derives from kidney and cardiovascular outcome evidence, not from demonstration that cytokine suppression mediates benefit. This distinction provides a useful model for interpreting other pleiotropic therapies.

#### 5.1.2. SGLT2 Inhibitors

SGLT2 inhibitors have transformed CKD care across diabetic and non-diabetic etiologies. In DAPA-CKD, dapagliflozin reduced the primary composite of sustained eGFR decline of at least 50%, kidney failure, or renal/cardiovascular death by 39% (hazard ratio [HR] 0.61, 95% confidence interval [CI] 0.51–0.72) [34]. In EMPA-KIDNEY, empagliflozin reduced kidney disease progression or cardiovascular death by 28% (HR 0.72, 95% CI 0.64–0.82) across a broader CKD population [35].

Mechanistically, SGLT2 inhibition reduces tubular workload, intraglomerular pressure, hypoxia, oxidative stress, and inflammatory signaling. Experimental studies suggest suppression of macrophage activation and NLRP3 signaling, including immunometabolic pathways involving tubular itaconate [36,37]. These observations strengthen biological plausibility but do not establish inflammatory mediation of the clinical benefit. Hemodynamic, metabolic, erythropoietic, and heart-failure effects operate concurrently.

#### 5.1.3. Finerenone

Mineralocorticoid receptor overactivation promotes sodium retention, endothelial dysfunction, macrophage recruitment, oxidative stress, and fibrosis. In FIDELIO-DKD, finerenone reduced the primary kidney composite in patients with type 2 diabetes and CKD (HR 0.82, 95% CI 0.73–0.93) [38]. The prespecified FIDELITY pooled analysis confirmed reductions in the cardiovascular composite (HR 0.86, 95% CI 0.78–0.95) and kidney composite (HR 0.77, 95% CI 0.67–0.88) [39]. Translational data support anti-inflammatory and antifibrotic actions [40], but, as with SGLT2 inhibitors, the trials were not designed to prove that inflammation was the causal mediator.

Combination therapy is clinically attractive because RAS blockade, SGLT2 inhibition, and finerenone act on complementary pathways. Available analyses suggest preserved finerenone benefit with background SGLT2 inhibitor use, but the number of participants receiving both agents in the original trials was limited [41]. Future mechanistic studies should determine whether combination therapy reduces residual inflammatory risk or primarily delivers additive hemodynamic and antifibrotic effects.

#### 5.1.4. GLP-1 Receptor Agonists

GLP-1RAs influence weight, glycemia, endothelial biology, macrophage phenotype, and inflammatory signaling. Experimental studies report attenuation of receptor for advanced glycation end-products and TLR4/MyD88/NF-κB pathways and modulation of adaptive immune activation [42,43,44]. The FLOW trial moved the class firmly into kidney outcome therapy: semaglutide reduced major kidney disease events by 24% (HR 0.76, 95% CI 0.66–0.88), cardiovascular death by 29%, and all-cause death by 20% in participants with type 2 diabetes and CKD [45].

The anti-inflammatory contribution to these outcomes remains uncertain because weight loss, glycemic improvement, blood pressure, natriuresis, and direct vascular effects are intertwined. GLP-1RAs should therefore be described as outcome-proven cardiorenal-metabolic drugs with anti-inflammatory actions, not as targeted anti-inflammatory agents.

### 5.2. IL-1 Pathway Inhibition: Proof of Principle Without CKD Outcome Confirmation

Canakinumab provided cardiovascular proof that selective anti-inflammatory therapy can reduce atherosclerotic events independently of lipid lowering. In the CANTOS CKD subgroup, participants with eGFR 30–60 mL/min/1.73 m^2^ experienced an 18% reduction in major adverse cardiovascular events (HR 0.82, 95% CI 0.68–1.00), with greater benefit among those achieving on-treatment hsCRP below 2 mg/L [46]. Canakinumab did not improve eGFR, and fatal infection was an important class-related safety concern in the parent trial.

In maintenance hemodialysis, an early pilot study of anakinra showed substantial reductions in hsCRP and IL-6 [47]. The subsequent ACTION pilot randomized 80 patients with hsCRP at least 2 mg/L to anakinra or placebo for 24 weeks. Anakinra was feasible and reduced IL-6, but the primary hsCRP endpoint was neutral and the study was not powered for cardiovascular events or mortality [48]. Collectively, these data establish biological activity and trial feasibility, but not routine clinical utility in CKD.

### 5.3. IL-6 Inhibition: Potent Target Engagement Without Demonstrated Outcome Benefit

IL-6 inhibition has generated the strongest human target-engagement signal. In the phase 2 RESCUE trial, 264 patients with CKD, established atherosclerotic cardiovascular disease, and hsCRP at least 2 mg/L received ziltivekimab or placebo. Median hsCRP fell by 77%, 88%, and 92% at 12 weeks with 7.5, 15, and 30 mg, respectively, compared with 4% on placebo; fibrinogen, serum amyloid A, secretory phospholipase A2, haptoglobin, and lipoprotein(a) also declined [49]. RESCUE-2 confirmed biomarker efficacy in a Japanese population [50].

These findings led to ZEUS (NCT05021835), an event-driven phase 3 cardiovascular outcome trial of monthly ziltivekimab 15 mg versus placebo in participants with atherosclerotic cardiovascular disease, CKD, and hsCRP at least 2 mg/L. The current registry lists 6385 enrolled participants, whereas the published baseline analysis described 6376 randomized participants; mean baseline eGFR was 44.5 mL/min/1.73 m^2^ and median hsCRP was 4.5 mg/L [51,52]. ZEUS was completed in June 2026. Sponsor-reported headline results released on 31 July 2026 showed no reduction in three-point MACE (HR 0.99, 95% CI 0.88–1.11) despite expected reductions in free IL-6 and hsCRP. Overall adverse-event and serious-adverse-event rates were similar, but serious infections were more frequent with ziltivekimab; all-cause mortality did not differ [53]. Full peer-reviewed results, including the prespecified kidney composite and subgroup analyses, were not available as of 16 August 2026.

Clazakizumab extends the IL-6 strategy to maintenance dialysis. In the phase 2b component of POSIBIL6ESKD, participants with cardiovascular disease and/or diabetes, dialysis dependence, and hsCRP at least 2 mg/L were randomized to clazakizumab 2.5, 5, or 10 mg or placebo every four weeks. Each active group included 32 participants and placebo included 31. At week 12, hsCRP fell by approximately 86–92%, and 79–82% of treated participants achieved hsCRP below 2 mg/L compared with none receiving placebo [54]. Secondary analyses suggested favorable effects on anemia and iron parameters and a reduction in neutrophil-to-lymphocyte ratio, further supporting pathway engagement [55,56].

Serious infections were numerically more frequent at the highest clazakizumab dose in phase 2b, emphasizing the need for dose selection and surveillance. POSIBIL6ESKD (NCT05485961) is a combined phase 2b/3 program. Part 1 enrolled 127 participants and established dose-dependent target engagement; Part 2 tests whether clazakizumab reduces cardiovascular death or myocardial infarction in dialysis patients with diabetes or atherosclerotic cardiovascular disease and hsCRP at least 2 mg/L [54,55,56,57]. The approximately 2190 figure reported in earlier protocol materials referred to the original phase 3 target rather than the entire program. The registry updated on 11 August 2026 now lists 3110 participants as the estimated combined enrollment and does not separately state the revised Part 2 target; recruitment remains ongoing [57]. After the neutral ZEUS result, POSIBIL6ESKD is not simply confirmatory: it tests a different IL-6 ligand antibody, dose, route, and dialysis-specific inflammatory phenotype, but infection and net clinical benefit require especially close evaluation.

### 5.4. Chemokine and JAK-STAT Inhibition

Human studies targeting inflammatory cell recruitment or downstream cytokine signaling have produced surrogate signals but have not progressed to outcome-proven CKD therapy. In a phase 2 trial in diabetic kidney disease, the JAK1/JAK2 inhibitor baricitinib reduced albuminuria, but safety concerns intrinsic to systemic JAK inhibition—including infection, cytopenia, and thrombosis—are particularly relevant in CKD [58]. The selective CCR2 antagonist CCX140-B reduced albuminuria by approximately 18% compared with 2% on placebo in type 2 diabetes and nephropathy, supporting the contribution of CCL2-mediated monocyte recruitment [59]. Neither program established an effect on kidney failure or cardiovascular outcomes.

These studies remain informative because they show that pathway-specific inflammation can modify a renal surrogate. They also illustrate why albuminuria reduction must not be treated as sufficient proof for immunomodulatory therapy: durability, off-target immune effects, and net clinical benefit require longer and larger trials.

### 5.5. NLRP3 Inhibition and Nrf2 Modulation

Direct NLRP3 inhibition is attractive because it lies upstream of IL-1β and IL-18 and integrates metabolic, crystal, mitochondrial, and uremic danger signals. MCC950 and CY-09 reduce inflammation, tubular injury, and fibrosis in experimental CKD models [14,60,61]. Human CKD development has now begun. AZD4144, an oral direct NLRP3 inhibitor, was evaluated in a randomized, double-blind, placebo-controlled phase 1b trial (NCT06675175) in 29 adults with established atherosclerotic cardiovascular disease, eGFR 30–59 mL/min/1.73 m^2^, and hsCRP above 2 mg/L. The 28-day study assessed safety and change in IL-6 as co-primary outcomes, with IL-18 and hsCRP as secondary pharmacodynamic measures; it was completed in October 2025, but results were not publicly posted as of 16 August 2026 [62]. Direct NLRP3 inhibition therefore remains Layer 3 until human safety and target-engagement data are reported and replicated; no inhibitor has demonstrated kidney or cardiovascular outcome benefit in CKD.

Nrf2 activation illustrates the difference between pathway rationale and clinical success. Bardoxolone methyl increased eGFR in earlier studies, yet the BEACON trial in stage 4 diabetic CKD was terminated because heart-failure hospitalization or death from heart failure was increased (HR 1.83, 95% CI 1.32–2.55), without reduction in kidney failure or cardiovascular death [63]. The mechanism likely involved acute sodium and volume retention in susceptible patients. The lesson is broader than one drug: therapies that alter filtration markers or antioxidant pathways require rigorous cardiovascular and volume-safety assessment.

### 5.6. Pentoxifylline, Colchicine, and Accessible Repurposing

Pentoxifylline inhibits phosphodiesterase activity and can reduce TNF-related signaling. In PREDIAN, adding pentoxifylline to RAS blockade in diabetic CKD slowed two-year eGFR decline and reduced albuminuria and urinary TNF [64]. Meta-analyses suggest favorable effects on inflammatory markers, albuminuria, and eGFR, but the evidence is limited by small samples, older background therapy, heterogeneity, and absence of definitive kidney-failure outcomes [65]. Pentoxifylline may be an accessible adjunct in selected settings, but it is not a substitute for contemporary standard therapy.

Low-dose colchicine reduces ischemic events in chronic coronary disease [66], but advanced CKD was underrepresented and CKD-specific inference remains limited. Colchicine has a narrow therapeutic window, interacts with CYP3A4 and P-glycoprotein inhibitors, and accumulates as kidney function declines; current cardiovascular guidance recommends dose adjustment or avoidance in severe CKD depending on indication and concomitant therapy [67]. Two prospective CKD programs are now relevant. CICI-HP (NCT05677555) is a recruiting phase 2 placebo-controlled study of colchicine 0.5 mg in 50 hemodialysis patients focused on inflammatory biomarkers [68]. RESOLVE-CKD (NCT07654231) is a not-yet-recruiting phase 2 pilot that will randomize 60 adults with stage 3 CKD, UACR at least 200 mg/g, and coronary calcification to low-dose colchicine plus usual care or usual care alone for 12 months; the primary endpoint is change in coronary artery calcium score, with CKD-mineral and bone disorder biomarkers and kidney measures as secondary or exploratory outcomes [69]. Neither trial is powered for kidney failure or cardiovascular events, so routine colchicine prescription solely for residual inflammatory risk in CKD remains unjustified.

### 5.7. Microbiota-Directed Interventions

Prebiotics, probiotics, synbiotics, resistant starch, and dietary strategies seek to reduce microbial generation of uremic toxins, restore short-chain fatty-acid production, and improve gut barrier integrity [70,71,72]. Meta-analysis of small randomized trials suggests reductions in CRP and oxidative-stress markers, but interventions, doses, CKD stages, diets, and outcome definitions are highly heterogeneous [71]. Effects on indoxyl sulfate and p-cresyl sulfate are inconsistent, and convincing evidence for slower CKD progression or fewer cardiovascular events is absent.

The microbiome remains an important upstream target, but future studies require standardized interventions, detailed dietary and medication phenotyping, metabolomic confirmation of target engagement, and clinically meaningful outcomes. Precision may be especially important because baseline microbiota composition and residual kidney function strongly influence response.

### 5.8. Comparative Therapeutic Evidence and Registered Pipeline

Table 1 and Table 2 compare therapeutic maturity and human evidence, whereas Table 3 summarizes all active or recently completed interventional trials identified by the updated registry search that met the prespecified CKD pipeline criteria. Detailed eligibility, endpoints, and exclusions are provided in Supplementary Table S1.

## 6. Toward Precision Anti-Inflammatory Nephrology

### 6.1. Inflammation Is an Endotype, Not a Diagnosis

The key therapeutic challenge is not finding a drug that lowers CRP; it is identifying patients in whom a specific inflammatory pathway is causal, active, and safely modifiable. CKD stage and albuminuria do not provide this information. Inflammatory endotyping should incorporate etiology, comorbidity, infection status, dialysis modality, vascular access, body composition, medication exposure, and longitudinal biomarkers.

At least three broad phenotypes can be distinguished conceptually. A tissue-dominant phenotype includes active immune or inflammatory kidney disease in which intrarenal pathways drive damage. A systemic metabolic-vascular phenotype includes diabetic or atherosclerotic CKD with persistent hsCRP/IL-6 elevation despite optimized standard therapy. A dialysis-amplified phenotype includes kidney failure with recurrent extracorporeal exposure, vascular access-related signals, wasting, and marked cardiovascular risk. These phenotypes may overlap, but they should not be assumed to respond identically.

### 6.2. Biomarkers for Enrichment and Response

hsCRP is inexpensive, standardized, and already used for enrichment in RESCUE, ZEUS, ACTION, and POSIBIL6ESKD. It is nevertheless downstream, nonspecific, and influenced by infection, obesity, access complications, and intercurrent illness. IL-6 is mechanistically closer to several relevant pathways and strongly prognostic, but assays and thresholds are less standardized [17,18,19]. Neutrophil-to-lymphocyte ratio is accessible and may reflect systemic inflammatory balance, but it is similarly nonspecific; the 2026 clazakizumab analysis supports pharmacodynamic responsiveness rather than validated predictive utility [56].

Urinary biomarkers may better reflect intrarenal activity. CCL2/MCP-1, TNF receptors, tubular injury markers, and urinary cytokines are plausible candidates, but few have been prospectively validated to select treatment. Gut-derived metabolites can characterize an upstream uremic-toxin phenotype, while multi-omics, immune-cell phenotyping, and single-cell tissue analysis may eventually identify pathway-specific signatures. For clinical implementation, a biomarker must do more than predict risk: it should identify differential treatment benefit or reliably confirm target engagement. These candidate tools are summarized in Table 4.

### 6.3. Safety Must Be Integrated into Biological Selection

Advanced CKD changes drug exposure and immune risk. Reduced renal clearance, altered protein binding, dialysis removal, and polypharmacy can narrow the therapeutic window. Before cytokine or inflammasome inhibition, protocols should explicitly address latent and active infection, vascular access, vaccination, cytopenia, liver disease, concomitant immunosuppression, and drug interactions. Repeated surveillance is required because inflammatory biomarkers can fall even when infection risk rises.

Dose selection should prioritize net benefit rather than maximal biomarker suppression. The numerical infection imbalance at the highest clazakizumab dose is a reminder that near-complete pathway inhibition may not be necessary or desirable [54]. Similarly, treatment interruption rules and adjudication of infection-related hospitalization should be integral trial components, not secondary safety details.

## 7. Priorities for the Next Generation of Trials

First, clinical outcomes must remain the decisive standard. ZEUS now provides a direct example: profound IL-6 pathway engagement did not reduce MACE, and serious infection was more frequent [53]. Biomarker and proteomic changes remain valuable for pharmacodynamic and mediation analyses, but they cannot substitute for cardiovascular events, kidney failure, sustained eGFR decline, infection-related hospitalization, or death.

Second, trials should test incremental benefit on top of contemporary CKD therapy and within a biologically coherent endotype. Earlier inflammatory studies predated widespread SGLT2 inhibitor, finerenone, and GLP-1RA use. Persistent inflammation after guideline-directed therapy may enrich risk, but ZEUS indicates that hsCRP enrichment alone does not guarantee causal responsiveness.

Third, repeated measurements should distinguish persistent inflammatory endotypes from transient elevations. A single hsCRP value may capture occult infection or an acute event. Run-in confirmation, exclusion of remediable sources, and longitudinal response thresholds can improve biological enrichment.

Fourth, kidney-specific and cardiovascular objectives should be separated when appropriate. A drug may reduce atherothrombotic events without altering intrinsic CKD progression, as suggested by CANTOS, or may reduce albuminuria without proven cardiovascular benefit, as in early JAK/CCR2 programs. Composite outcomes should not obscure these distinct biological questions.

Fifth, mechanistic trials should incorporate tissue, imaging, and multi-omic substudies without allowing complexity to impede clinical interpretability. Prespecified mediation analyses can test whether changes in IL-6, hsCRP, fibrinogen, anemia, or immune-cell signatures account for outcome effects. Such analyses will help determine whether a biomarker is merely prognostic, pharmacodynamic, or truly on the causal pathway.

Finally, implementation and cost must be considered. Monoclonal antibodies may be justified in a highly enriched, high-risk population if they reduce major events, but broad use would require durable safety, feasible monitoring, and health-economic value. Lower-cost approaches such as dietary modification, optimized dialysis practice, or repurposed drugs remain attractive, yet they require the same evidentiary discipline.

## 8. Conclusions

Inflammation is neither an incidental laboratory abnormality nor a single therapeutic target in CKD. It is a heterogeneous network connecting tissue injury, uremic retention, oxidative and metabolic stress, gut barrier failure, maladaptive immunity, fibrosis, vascular disease, and premature aging. This network helps explain residual kidney and cardiovascular risk, but it does not justify indiscriminate immunosuppression.

The therapeutic landscape is best understood as a continuum. RAS inhibitors, SGLT2 inhibitors, finerenone, and GLP-1RAs have proven cardiorenal benefits and plausible anti-inflammatory actions, although those actions have not been established as the dominant mechanism of benefit. IL-1 blockade provides proof of principle. IL-6 inhibition achieves unusually strong target engagement, but ZEUS demonstrated that this is insufficient for MACE reduction in an ASCVD/CKD population and reinforced the infection trade-off; POSIBIL6ESKD remains an independent dialysis-specific outcome test. JAK/CCR2 inhibition, direct NLRP3 blockade, Nrf2 modulation, and microbiota-directed strategies remain earlier in translation or constrained by safety and evidentiary limitations.

The field will advance if it moves from the broad label of “inflammatory CKD” to reproducible, pathway-aligned endotypes and treats neutral trials as mechanistically informative. ZEUS defines an important boundary: biomarker enrichment and suppression did not establish cardiovascular benefit. POSIBIL6ESKD, early NLRP3 programs, and CKD-specific colchicine trials will test different populations and endpoints, but the standard should remain net clinical benefit beyond optimized contemporary treatment without worsening infection, toxicity, or frailty risks intrinsic to CKD.

## Figures and Tables

**Table 1:** Anti-inflammatory therapeutic strategies in CKD according to translational maturity.

Strategy	Principal Inflammatory Leverage	Highest Level of CKD-Relevant Evidence	Main Limitation	Current Interpretation
RAS inhibitors	Angiotensin II-driven oxidative and inflammatory signaling	Outcome-proven renoprotection; supportive biomarker effects [3,33]	Inflammation is not an established mediator	Foundational therapy
SGLT2 inhibitors	Tubular immunometabolism, oxidative stress, macrophage and NLRP3 signaling	Large kidney outcome trials across diabetic and non-diabetic CKD [34,35,36,37]	Mechanistic effects are pleiotropic	Cornerstone cardiorenal therapy
Finerenone	Mineralocorticoid receptor-driven inflammation and fibrosis	Kidney and cardiovascular outcome benefit in type 2 diabetes with CKD [38,39,40]	Hyperkalemia; evidence strongest in diabetic CKD	Outcome-proven add-on
GLP-1RAs	Metabolic, endothelial, macrophage, and NF-κB modulation	FLOW kidney and cardiovascular outcome benefit [42,43,45]	Evidence concentrated in type 2 diabetes	Outcome-proven cardiorenal-metabolic therapy
IL-1 blockade	IL-1-dependent cytokine amplification	CANTOS CKD subgroup; dialysis pilot trials [46,47,48]	No definitive CKD outcome trial; infection risk	Proof of principle
IL-6 blockade	Acute-phase, endothelial, thrombotic, and anemia pathways	Profound target engagement; ZEUS phase 3 neutral for MACE; POSIBIL6ESKD ongoing [49,50,51,52,53,54,55,56,57]	No demonstrated hard-outcome benefit; infection signal	Advanced Layer 2 strategy, not established therapy
JAK1/2 or CCR2 inhibition	Cytokine signal transduction or monocyte recruitment	Phase 2 albuminuria reduction [58,59]	Surrogate outcomes; systemic immune toxicity	Developmental evidence
NLRP3 inhibition	Upstream inflammasome activation, IL-1β/IL-18 maturation, and pyroptosis	Experimental renoprotection; phase 1b CKD program completed without public results [14,60,61,62]	No reported CKD efficacy or outcome evidence	Early human development (Layer 3)
Nrf2 activation	Antioxidant and cytoprotective transcription	Human efficacy signal offset by cardiovascular harm in BEACON [63]	Fluid retention and cardiovascular safety	Cautionary precedent
Pentoxifylline	TNF-related and hemorheologic effects	Small trials and heterogeneous meta-analyses [64,65]	Older background therapy; no hard outcomes	Optional/adjunctive
Colchicine	Microtubule-dependent innate immune and inflammasome effects	Cardiovascular benefit outside advanced CKD; two small CKD trials registered [66,67,68,69]	Accumulation, interactions, neuromyotoxicity; no hard CKD outcomes	Investigational in CKD
Microbiota-directed therapy	Toxin generation and gut barrier dysfunction	Small heterogeneous biomarker trials [70,71,72]	No consistent kidney or cardiovascular outcomes	Investigational adjunct

**Table 2:** Selected completed or reporting human studies of targeted anti-inflammatory therapy relevant to CKD.

Agent/Pathway	Study and Population	Principal Finding	What the Study Establishes	What Remains Unknown
Canakinumab/IL-1β	CANTOS CKD subgroup; prior myocardial infarction, eGFR 30–60 mL/min/1.73 m^2^	MACE HR 0.82; greater benefit in hsCRP responders [46]	Cardiovascular proof of inflammatory causality in CKD-adjacent disease	Kidney benefit; net benefit in broader CKD
Anakinra/IL-1 receptor	ACTION; 80 hemodialysis patients with hsCRP ≥2 mg/L	Feasible; IL-6 reduced; primary hsCRP endpoint neutral [48]	Dialysis feasibility and biological activity	Cardiovascular or mortality benefit
Ziltivekimab/IL-6 ligand	RESCUE; 264 patients with CKD, ASCVD, and inflammation	Dose-dependent hsCRP reduction of 77–92% with broad biomarker effects [49]	Robust human target engagement	Clinical outcomes
Ziltivekimab/IL-6 ligand	ZEUS; NCT05021835; phase 3 ASCVD, CKD, hsCRP ≥ 2 mg/L; registry n = 6385	Completed; headline MACE HR 0.99 (95% CI 0.88–1.11); target engagement preserved; serious infections more frequent [51,52,53]	Biomarker suppression did not translate into MACE benefit	Full peer-reviewed results, kidney outcome, and subgroup effects
Clazakizumab/IL-6 ligand	POSIBIL6ESKD phase 2b; 127 dialysis patients	hsCRP reduction of approximately 86–92%; anemia and NLR signals [54,55,56]	Potent target engagement in dialysis	Cardiovascular benefit and infection trade-off
Clazakizumab/IL-6 ligand	POSIBIL6ESKD Part 2; NCT05485961; inflammatory ESKD on dialysis	Recruiting; combined program enrollment currently estimated at 3110 [57]	Direct phase 3 test of CV death or MI in dialysis	Efficacy, infection trade-off, and net clinical benefit
Baricitinib/JAK1/2	Phase 2 diabetic kidney disease trial	Albuminuria reduction [58]	Surrogate kidney effect	Hard outcomes and CKD-specific safety
CCX140-B/CCR2	Phase 2 type 2 diabetes with nephropathy	Albuminuria reduced by approximately 18% versus 2% with placebo [59]	Role of monocyte recruitment in a human surrogate	Kidney failure and cardiovascular outcomes

**Table 3:** Active or recently completed registered interventional trials meeting the CKD anti-inflammatory pipeline criteria (registry information verified 16 August 2026).

Study/Agent	Phase and Design	Target Population	N	Primary and Key Secondary Outcomes	Status and Available Evidence
ZEUS/ziltivekimab NCT05021835	Phase 3; randomized, double-blind, placebo-controlled; monthly 15 mg SC	ASCVD plus CKD and hsCRP ≥ 2 mg/L; active infection excluded	6385 registry; 6376 published baseline cohort	Primary: 3-point MACE. Key secondary: expanded MACE, HF outcome, mortality, kidney composite	Completed June 2026. Headline MACE neutral (HR 0.99); biomarker target engagement; more serious infections; full results pending [51,52,53]
POSIBIL6ESKD/clazakizumab NCT05485961	Combined phase 2b/3; randomized, double-blind, placebo-controlled; IV every 4 weeks	Maintenance dialysis ≥ 12 weeks, hsCRP ≥ 2 mg/L, diabetes or ASCVD	3110 estimated combined enrollment	Part 2 primary: time to CV death or MI. Secondary: mortality, HF and CV hospitalizations, safety	Recruiting. Part 1 target engagement reported; no Part 2 outcomes [54,55,56,57]
AZD4144 NCT06675175	Phase 1b; randomized, double-blind, placebo-controlled; oral treatment 28 days	ASCVD, eGFR 30–59 mL/min/1.73 m^2^, hsCRP > 2 mg/L; dialysis/transplant excluded	29 actual	Co-primary: adverse events and relative IL-6 change. Secondary: IL-18, hsCRP, pharmacokinetics	Completed October 2025; no public results posted [62]
CICI-HP/colchicine NCT05677555	Phase 2; randomized, placebo-controlled; colchicine 0.5 mg	Adults receiving maintenance hemodialysis with chronic low-grade inflammation	50 estimated	Inflammatory biomarker change; safety and tolerability	Recruiting; no results [68]
RESOLVE-CKD/low-dose colchicine NCT07654231	Phase 2 pilot; randomized, open-label, outcome-blinded; 12 months	Stage 3 CKD, UACR ≥ 200 mg/g, CAC score ≥ 30, plus CV risk or ASCVD	60 estimated	Primary: change in CAC Agatston score. Secondary/exploratory: CKD-MBD markers, UACR, eGFR, ABI/TBI, safety	Not yet recruiting; no results [69]

**Table 4:** Candidate tools for inflammatory enrichment in CKD trials.

Candidate	Strength	Major Limitation	Most Appropriate Current Use
hsCRP	Standardized, inexpensive, repeated measurement feasible	Downstream and nonspecific	Trial enrichment and pharmacodynamic response
Circulating IL-6	Mechanistically aligned with leading targeted programs	Assay variability; affected by infection and comorbidity	Pathway characterization and exploratory prediction
Neutrophil-to-lymphocyte ratio	Universally available and low cost	Strongly affected by infection, corticosteroids, and hematologic factors	Exploratory risk and pharmacodynamic marker
Urinary CCL2/MCP-1	Potentially reflects intrarenal monocyte recruitment	Depends on albuminuria, urine concentration, and etiology	Mechanistic trials of chemokine-directed therapy
TNF receptors and tubular injury markers	Strong prognostic associations in several CKD cohorts	Predictive value for anti-inflammatory treatment unproven	Risk stratification and composite endotyping
Uremic toxin/metabolomic profile	Links gut metabolism, clearance, and vascular biology	Limited standardization and strong dietary dependence	Microbiota and toxin-targeted studies
Immune-cell or transcriptomic signatures	Greater pathway specificity	Cost, complexity, and limited external validation	Early-phase precision trials

## Data Availability

No new data were created or analyzed in this study. Data sharing is not applicable to this article.

## Supplementary Materials

The following supporting information can be downloaded at: https://www.mdpi.com/article/10.3390/jcm15176550/s1, Supplementary Methods S1: Complete PubMed/MEDLINE and ClinicalTrials.gov search strategies and study-selection criteria; Table S1: Detailed characteristics of active or recently completed registered interventional trials.
