# Supplementary material for: Targeting Inflammation in Chronic Kidney Disease: Pathophysiological Insights and Emerging Therapeutic Strategies
Source: J Clin Med. 2026 Aug 25;15(17):6550. doi: 10.3390/jcm15176550 (PMC13566433; doi:10.3390/jcm15176550)
Supplement: Supplementary file 1 [file jcm-15-06550-s001.zip › jcm-4495636-supplementary.pdf]

Supplementary Material

Targeting Inflammation in Chronic Kidney Disease: Pathophysiological Insights and Emerging Therapeutic Strategies

Supplementary Methods S1. Literature and Trial-Registry Search

The searches were performed until July 2026. The purpose was transparent, targeted identification for a narrative review rather than exhaustive systematic evidence synthesis. Searches were restricted to English-language records. Reference lists of key guidelines, randomized trials, and recent high-quality reviews were screened manually. Trial identifiers and registry fields were verified immediately before revision.

PubMed/MEDLINE - complete search string

(("Kidney Diseases, Chronic"[Mesh] OR "chronic kidney disease"[Title/Abstract] OR CKD[Title/Abstract] OR "renal insufficiency, chronic"[Title/Abstract] OR dialysis[Title/Abstract] OR "kidney failure"[Title/Abstract]) AND ("Inflammation"[Mesh] OR inflamm*[Title/Abstract] OR "innate immunity"[Title/Abstract] OR NLRP3[Title/Abstract] OR inflammasom*[Title/Abstract] OR "interleukin-1"[Title/Abstract] OR "IL-1"[Title/Abstract] OR "interleukin-6"[Title/Abstract] OR "IL-6"[Title/Abstract] OR "oxidative stress"[Title/Abstract] OR "uremic toxin"[Title/Abstract] OR "uremic toxins"[Title/Abstract] OR microbiota[Title/Abstract] OR "gut-kidney axis"[Title/Abstract]) AND (therap*[Title/Abstract] OR treat*[Title/Abstract] OR trial*[Title/Abstract] OR inhibitor*[Title/Abstract] OR antagonist*[Title/Abstract] OR "SGLT2 inhibitor"[Title/Abstract] OR finerenone[Title/Abstract] OR "GLP-1 receptor agonist"[Title/Abstract] OR baricitinib[Title/Abstract] OR "CCX140-B"[Title/Abstract] OR pentoxifylline[Title/Abstract] OR colchicine[Title/Abstract] OR ziltivekimab[Title/Abstract] OR clazakizumab[Title/Abstract] OR AZD4144[Title/Abstract]))

ClinicalTrials.gov - condition/disease field

"Chronic Kidney Disease" OR "Kidney Failure" OR "End Stage Renal Disease" OR Dialysis

ClinicalTrials.gov - other terms field

inflammation OR inflammatory OR NLRP3 OR inflammasome OR "interleukin-1" OR "IL-1" OR "interleukin-6" OR "IL-6" OR JAK OR CCR2 OR colchicine OR pentoxifylline OR microbiome OR ziltivekimab OR clazakizumab OR AZD4144

ClinicalTrials.gov filters: Interventional Studies; Adult and Older Adult; all recruitment statuses. A second named-agent search (ziltivekimab, clazakizumab, anakinra, canakinumab, baricitinib, CCX140-B, AZD4144, pentoxifylline, and colchicine) was performed to identify records with incomplete condition indexing.

Eligibility and Selection Rules

- Population: adults with non-immune CKD, kidney failure receiving maintenance dialysis, or CKD-enriched cardiovascular disease.
- Intervention: direct inflammatory-pathway therapy or established cardiorenal therapy with a clinically relevant anti-inflammatory mechanism.
- Evidence priority: randomized clinical outcomes, randomized target engagement, prospective interventional registry records, systematic reviews, and high-quality mechanistic studies.
- Preclinical studies: included only where CKD-specific human efficacy data were unavailable.
- Excluded from the therapeutic pipeline: acute kidney injury; disease-specific immunosuppression for active glomerulonephritis or transplantation; observational biomarker studies without therapeutic evaluation; non-CKD trials without direct translational relevance; and duplicate or superseded registry records.
- Screening: both authors reviewed candidate records and agreed on final inclusion by consensus. No formal risk-of-bias tool or PRISMA flow diagram was used because this was a targeted narrative review.

Supplementary Table S1

Detailed characteristics of active or recently completed registered interventional trials meeting the CKD anti-inflammatory pipeline criteria. Registry data were verified until July 2026.

Study / mechanism	Phase / design	Population and key inclusion	Key exclusions	N	Primary endpoint	Key secondary endpoints	Status	Available efficacy / safety
ZEUS<br>Ziltivekimab; IL-6 ligand<br>NCT05021835	Phase 3; randomized, double-blind, placebo-controlled; monthly SC 15 mg; event-driven	Adults with established ASCVD; CKD defined by eGFR 15-<60 or UACR ≥200 mg/g with eGFR ≥60; hsCRP ≥2 mg/L	Active infection; recent MI/stroke/unstable angina/TIA; planned revascularization or major surgery	6385 registry; 6376 baseline publication	Time to 3-point MACE: CV death, non-fatal MI, or non-fatal stroke	Expanded MACE; HF outcome; all-cause death; kidney composite; inflammation and severe infection	Completed June 2026	Headline: MACE HR 0.99 (95% CI 0.88-1.11); IL-6/hsCRP reduced; serious infections more frequent; mortality similar. Full peer-reviewed and kidney results pending.
POSIBIL6ESKD<br>Clazakizumab; IL-6 ligand<br>NCT05485961	Combined phase 2b/3; randomized, quadruple-blind, placebo-controlled; IV every 4 weeks	Age ≥18; maintenance dialysis ≥12 weeks; hsCRP ≥2 mg/L; diabetes or ASCVD	Systemic immunosuppression; abnormal liver tests; active/opportunistic infection; limited life expectancy; GI perforation/IBD/ulcer disease; Part 1 participation excludes Part 2	3110 estimated combined enrollment; Part 1 enrolled 127	Part 1: log hsCRP change to week 12. Part 2: time to CV death or MI	Mortality; HF/CV hospitalization; limb events; total hospitalizations; biomarker and safety outcomes	Recruiting; current registry updated 11 August 2026	Part 1: hsCRP reduction approximately 86-92%; highest dose had numerically more serious infections. No Part 2 outcome data.
AZD4144<br>Direct NLRP3 inhibitor<br>NCT06675175	Phase 1b; randomized 1:1, double-blind, placebo-controlled; oral 28 days; follow-up to day 56	Age 18-80; established ASCVD; eGFR 30-<60; hsCRP >2 mg/L; BMI 18-45 kg/m²	Recent ASCVD event; active/chronic/recurrent infection; dialysis or transplant; recent AKI; recent malignancy	29 actual	Adverse events and relative change in systemic IL-6 at 4 weeks	IL-18 and hsCRP change; pharmacokinetics	Completed October 2025	No public result record or peer-reviewed report available as of access date.
CICI-HP<br>Colchicine; microtubule/innate immune modulation<br>NCT05677555	Phase 2; randomized, placebo-controlled; colchicine 0.5 mg	Adults receiving maintenance hemodialysis with chronic low-grade inflammation	Registry-specific safety exclusions apply; detailed current record should be rechecked before enrollment	50 estimated	Change in inflammatory biomarkers	Safety and tolerability	Recruiting	No results available.
RESOLVE-CKD<br>Colchicine; microtubule/innate immune modulation<br>NCT07654231	Phase 2 pilot; randomized, open-label, outcome-blinded; colchicine 0.5 mg daily plus usual care vs usual care; 12 months	Age 18-69; eGFR 30-59; UACR ≥200 mg/g; CAC score ≥30; hypertension, diabetes, dyslipidemia, or established ASCVD	Current colchicine; eGFR <30; recent infection/hospitalization; immunosuppression/cancer; pregnancy; strong CYP3A4 or P-gp inhibitors; severe anemia	60 estimated	Change in CAC Agatston score from baseline to 12 months	CKD-MBD and vascular calcification markers; UACR, eGFR, ABI/TBI; safety	Not yet recruiting	No results available.

Abbreviations: ABI, ankle-brachial index; ASCVD, atherosclerotic cardiovascular disease; CAC, coronary artery calcium; CKD-MBD, chronic kidney disease-mineral and bone disorder; CV, cardiovascular; HF, heart failure; hsCRP, high-sensitivity C-reactive protein; MACE, major adverse cardiovascular events; MI, myocardial infarction; P-gp, P-glycoprotein; SC, subcutaneous; TBI, toe-brachial index; TIA, transient ischemic attack; UACR, urine albumin-to-creatinine ratio.
